# The Final Frontier: Palliative Care in Space is an Inevitability

**DOI:** 10.1177/08258597231159839

**Published:** 2023-02-27

**Authors:** Sheryn Tan, Vienna Tran, Brandon Stretton, Aashray Gupta, Joshua Kovoor, Stephen Bacchi

**Affiliations:** 1 The University of Adelaide, Adelaide Medical School, Adelaide, SA, Australia; 2 Royal Adelaide Hospital, Adelaide, SA, Australia; 3 Flinders University, Bedford Park, SA, Australia

**Keywords:** pain, symptom control, end-of-life, spaceflight, astronaut

## Abstract

As space exploration becomes increasingly common, palliative care for astronauts will require greater consideration. All aspects of palliative care need to be specifically adapted for astronauts. For example, addressing additional circumstances such as inability to see loved ones from Earth will be an important part of meeting their psychological and spiritual needs. A different approach to pharmacological management of end-of-life symptoms is also warranted due to changes in human physiology and pharmacokinetics in space.

Providing medical care for astronauts presents unique challenges.^1^ Extensive research has been conducted on maintaining health of astronauts.^2^ In recent years, there has been increased government, commercial, and public interest in sending more humans to space, for longer time periods.^3,4^ As “one-way trips” to other celestial bodies become more realistic,^5^ it is inevitable that at some stage individuals will die while in space. Accordingly, palliative care for astronauts deserves deliberate consideration.

Palliative care focuses on symptom relief rather than prolonging life for patients with life-limiting illness.^6^ It is generally accepted to include five phases: stable, unstable, deteriorating, terminal and bereavement.^7^ While all phases will require specific consideration, two areas that will be particularly challenging are addressing the psychological and spiritual needs during deteriorating phase and the pharmacological management of symptoms during the terminal phase.

The psychological and spiritual health of astronauts is critical for crew health and achieving mission objectives. The unique challenges of the space environment, including prolonged confinement, isolation and distance from Earth, can all impact safety and interpersonal dynamics among crew.^8^ Similar effects have been observed during quarantine in the COVID-19 pandemic.^9^ Many doctors can attest to how difficult aspects of palliative care were during the pandemic. Palliative care physicians described feelings of distress and helplessness in instances such as when patients had to die alone owing to the unpredictable prognosis of COVID-19, or when family members were denied of patient contact.^10,11^ Comparably, individuals who deteriorate in the confined environment of space are likely to have unique psychological challenges, including the inability to see loved ones, and difficulties of receiving counselling. While telehealth options may be available, electronic communications may be significantly delayed due to interplanetary distances.^12^

The pharmacological management of many conditions, including the terminal phase of life, is also proposed to change in space. Many aspects of human physiology change due to altered gravity, which subsequently affect pharmacokinetics.^13^ Additionally, the systems and equipment used to administer medications on Earth may not be as effective in space. For example, since there is no separation between gas and liquid in microgravity, pumps must be used to deliver intravenous medications to decrease risk of air emboli.^14^ Another concern is the shorter shelf life of medications, largely due to greater amount of radiation outside the Earth's protective magnetosphere.^15^ To preserve the efficacy of critical medications during long-duration missions, various strategies have been proposed, including developing formulations less susceptible to radiation, or synthesising the medications in situ rather than relying on Earth's supply.^16,17^

Work within space medicine should explicitly consider palliative care needs of astronauts. Although largely theoretical at this stage, current strategies to provide palliative care in space may include medications that have been investigated and utilised in space medicine previously including analgesic agents (such as hydromorphone), antiemetics (such as dexamethasone, lorazepam and promethazine) and anxiolytics (such as lorazepam).^18,19^ Telemedicine strategies, including exploratory three-dimensional projection-based technology,^20^ may be used to help meet psychosocial and spiritual needs. However, further research is needed to help alleviate symptoms for an individual dying in space. This research may focus on pharmacological management strategies (such as how best to deliver continuous infusions in a palliative setting), non-pharmacological management (such as safety considerations for terminal seizures in zero gravity), and technological approaches (including the optimisation of meaningful interaction with relatives through telecommunication technologies). Additionally, consideration should be given for potentially new end-of-life syndromes that may develop in space, such as the manifestations of spaceflight-associated neuro-ocular syndrome and musculoskeletal deconditioning due to decreased weightbearing.^21^ The effect of bereavement on family members on Earth, along with other astronauts, should also be considered.

Palliative care for astronauts is relevant to space exploration. Astronauts requiring palliative care will require a unique approach to meeting their psychological and spiritual needs and optimising pharmacological management of end-of-life symptoms.
